# COVID-19: Medical education from the point of view of medical students using the participatory Delphi method

**DOI:** 10.1371/journal.pone.0297602

**Published:** 2024-07-05

**Authors:** Andrea Gabriela Ortiz Riofrio, Emilia José Valdivieso-Andrade, Nathaly Monserrath Acosta Masaquiza, Alex S. Aguirre, Nicolás Alexander Almeida Villavicencio, Cynthia Samantha Calderón Pilla, Prisca Del Pozo Acosta, Auki Guaillas Japón, Darwin Vicente Luna Chonata, Navila Bianca Mafla Roca, Alissa Solange Mendoza García, Lenin Andrés Muñoz Caicedo, Gustavo Alexander Muñoz Salazar, Kimberly Mishell Pacheco Reinoso, Camila Nicole Pazmiño Chávez, Nuria Karina Proaño Lozada, Jonathan Rzonzew Sauer, Gianny Alexander Saldaña Armas, Ivonne Estefania Salinas Avalo, Ana Cristina Saltos Granizo, Bonny Francisca Soria Sarabia, Doménica Alejandra Suárez Morales, Rodrigo Felipe Sulca Caillagua, María Antonia Zavala Cárdenas, Flavio Carrera Verdesoto, Diego Cisneros-Heredia, Pablo Estrella Porter, Jonathan Raymond Guillemot

**Affiliations:** 1 Escuela de Medicina, Universidad San Francisco de Quito USFQ, Quito, Ecuador; 2 Instituto de Medicina Social & Desafíos Globales, Universidad San Francisco de Quito USFQ, Quito, Ecuador; 3 Programa UNIDiversidad, Universidad San Francisco de Quito USFQ, Quito, Ecuador; 4 Instituto de Estudios Avanzados en Desigualdades, Colegio de Ciencias Sociales y Humanidades, Universidad San Francisco de Quito USFQ, Quito, Ecuador; 5 Laboratorio de Ecología Urbana y Rural, Instituto IBIOTROP, Colegio de Ciencias Biológicas y Ambientales, Universidad San Francisco de Quito USFQ, Quito, Ecuador; 6 Laboratorio de Salud Animal, Escuela de Medicina Veterinaria, Instituto IBIOTROP, Hospital de Fauna Silvestre TUERI, Universidad San Francisco de Quito USFQ, Quito, Ecuador; 7 Departamento de Medicina Preventiva y Salud Pública, Hospital Clínico Universitario de Valencia, Valencia, Spain; Lahore Medical and Dental College, PAKISTAN

## Abstract

**Background:**

The COVID-19 pandemic has prompted a transformation of medical training. Although there were obvious medical education and social interaction challenges, e-learning presented some advantages, which may have generated medical curricula innovation and adjustments to novel technological methodologies. This study aims to generate consensuses among medical students regarding medical education provided during the pandemic in the resource-limited context of a Global South university.

**Methods:**

The implementation of a participatory Delphi method included a recruitment campaign, training, constitution of Delphi panels and questions, and development of the Delphi exercises. Students from the second to the sixth year of medicine of a university in Quito, Ecuador, constituted two Delphi panels, developed questions about the education received during the pandemic, and answered them over 3.5 rounds.

**Findings:**

Twenty-two medical students participated in the Delphi exercises about their perception of medical education during the COVID-19 pandemic. The analysis consisted of a total of 22 Delphi questions divided into five distinct categories: adaptations and innovations, curriculum and assessment changes, virtual clinical practice, time management, and mental health. The authors established high, medium, and low consensuses for analysis.

**Conclusions:**

Consensuses were reached based on students’ academic year and focused on the changes in lecture delivery, the usage of new technologies, patient care skills, the impact of the educational routine, and the mental health of the COVID-19 pandemic. The way the pandemic affected medical education in the Global South set the stage for the need for a comprehensive review of tools, skills, and curricula for students from culturally diverse backgrounds. This study offers a highly replicable methodology to generate consensuses and introduce students to academic research.

## Introduction

The COVID-19 global pandemic challenged medical training. With the emergence of isolation, a new education model, including remote interactions between lecturers, students and patients, unexpectedly replaced the learning model of medicine of in-person lectures, clinical rotations, and face-to-face patient interactions [1, 2]. A wealth of technological tools was quickly incorporated, including web conferencing platforms, virtual patient simulators, and remote medical learning platforms allowing synchronous and asynchronous education [3]. Yet, the effect of e-learning tools has been heterogeneous among medical students. A study reported that preclinical students, whose main learning strategy was lecture-based, experienced a lower impact during the pandemic but were expected to possibly undergo training difficulties during future clinical years [4]. Clinical students, on the other hand, had restricted access to hands-on experience with limited exposure to clinical environments and little development of interpersonal skills, such as empathy and physical examination [1].

During the pandemic, frequent and persistent issues in the academic environment included defective or low internet speed, streaming malfunctions, lecturers and students lacking technical skills, and limited access to technological tools, among others. These concerns were more relevant in countries of the Global South, such as Ecuador, where medical students perceived an overall negative impact on their training [5]. Despite these challenges, e-learning presented advantages, including time flexibility, asynchronous and autonomous learning, and remote access to world-class medical knowledge [6]. The impact of COVID-19 continues to influence medical education, even as the pandemic seems to stabilize. Professors and students face challenges trying to establish a new normal for face-to-face education by implementing strict biosecurity measures, such as face masks, vaccination programs, and classes in hybrid- modality classes [7].

In Andean countries, little research includes medical students to provide solutions and policy changes, especially when considering the impact of the COVID-19 pandemic [8, 9]. Using a student-led participatory approach, we aimed to generate consensus regarding the lessons for medical education following the e-learning experience associated with COVID-19 at a university in Ecuador. This research created a space to listen to the perspectives of medical students of the Global South and contributed to a diagnostic evaluation of their learning process. Participants of this research represent a multicultural sample of students whose expertise in e-learning in times of COVID-19 could positively contribute to the creation and implementation of updated curricula, responding to their particular needs and influencing their successful professional training.

## Materials and methods

We developed and conducted two participatory Delphi panels with twenty-two medical students from the Universidad San Francisco de Quito USFQ, a private liberal-arts university in Quito, Ecuador, to generate consensus regarding ideas and opinions about medical e-learning during the COVID-19 pandemic.

A Delphi panel is a qualitative consensus-building methodology whereby experts use iterations to provoke the convergence of ideas [10]. A Delphi exercise unites 8–20 experts who respond to an open-ended Delphi questionnaire [11]. Questions for exercises are adequate when their answers refer to the panelist’s experience and are neither sufficiently informed by scientific facts nor based only on belief. The Delphi approach in the field of health is frequently applied for clinical guidelines development [12]. In the context of this study, medical students are considered the expert population based on their unique perspective on medical education during the COVID-19 pandemic. Additionally, participatory research addresses research questions with the active involvement of the study population, whereby participants are trained in the research methods, which often includes collecting the data and contributing to all or several elements of the research conduct [13].

We chose a participatory approach to enable students to be researchers and study subjects simultaneously. By not involving researchers who are also professors of the students, the authority barriers and risk of response bias were minimized. A Delphi study conducted previously at USFQ demonstrated the feasibility and effectiveness of this approach [8]. The campaign to recruit participants for the Delphi panels ‘S1 Video’ and the training session ‘S2 Video’ took place in September 2021. Approximately one hundred USFQ medical students registered for the training. To be eligible, students had to be attending the USFQ School of Medicine ‘S1 File’ undergoing at least their second year of studies, have no record of breach of the academic honor code ‘S2 File’ and to take a virtual examination with a minimum pass score of 7/10 ‘S3 File’. Twenty-four students met all inclusion criteria and desired acting as panelists. Two students were excluded for a history of breach of the USFQ code of honor. We formed two Delphi panels based on the academic year of participants. Each panel met virtually and independently to discuss the topics which they felt were relevant to their experience and develop the Delphi questionnaire. It is worth considering that the questions themselves constituted results as they illustrated the fields in which students felt competent and permitted the identification of relevant areas for consensus development.

**Fig 1:**
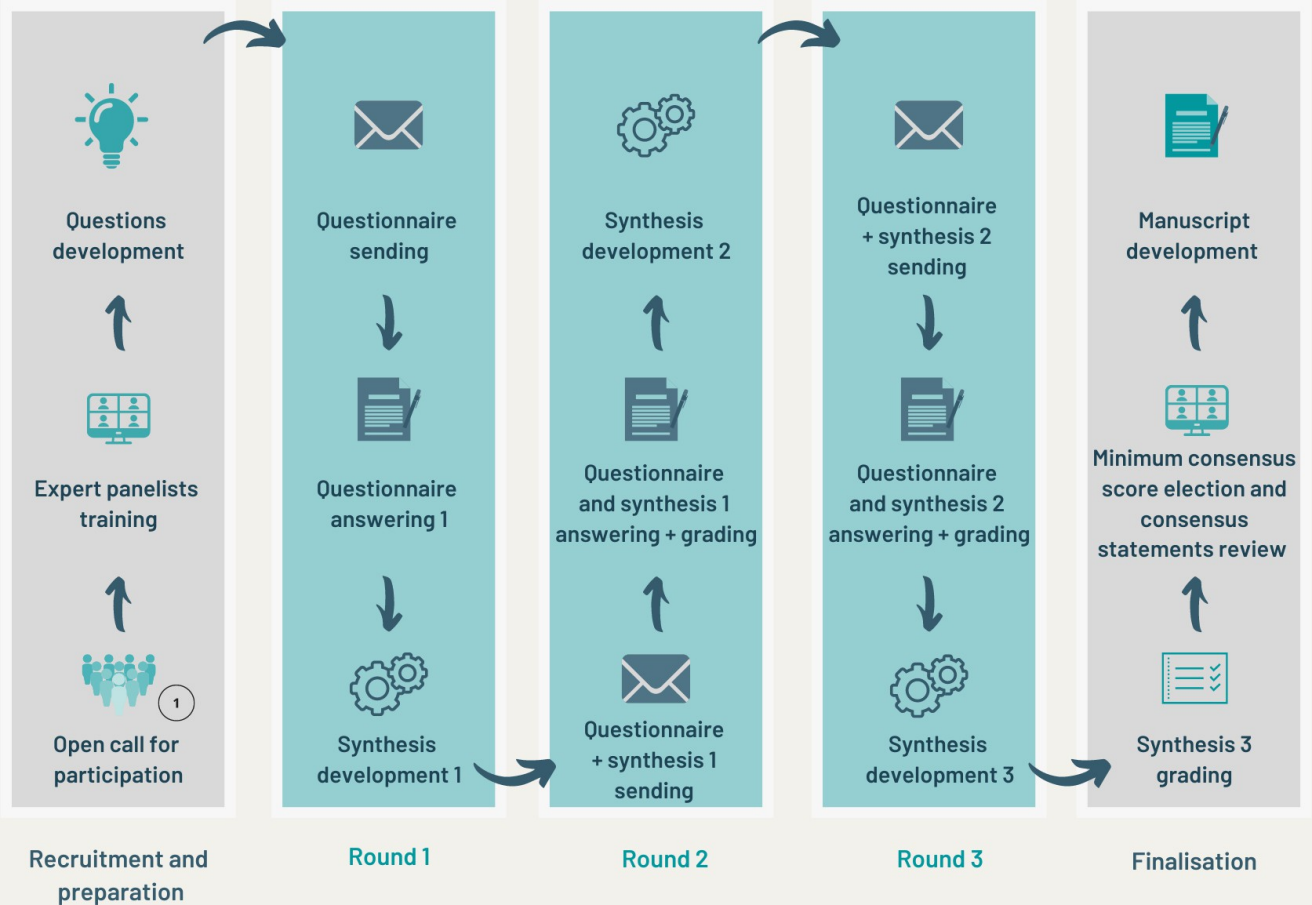
Graphic representation of Delphi methodology implemented by medicine panels. The study protocol 2021-119IN was certified exempt from a full ethical board review by the Ethics Committee of Universidad San Francisco de Quito (CE 054-2021-P21.119 IN-CEISH-USFQ).

The Delphi exercises consisted of 3.5 rounds as explained in Fig 1. A synthesis, developed for each question after each round, is a text that aims to turn questionnaire answers into potential consensus statements. All panelists then rated each synthesis on a scale from 0–10, based on how much they could agree with it. Scores were averaged for each round, with 10 representing complete agreement. As each round was followed by an evaluation by each panelist, a half round was added to allow the evaluation of the third round, hence a total of 3.5 rounds. Three categories of consensuses for the final result analysis were used: higher consensus (>9/10), mid consensus (>7–9/10) and lower consensus (>5–7/10). The exercises were conducted in Spanish, and translated excerpts in English are provided in this manuscript. We categorized all questions of Delphi panels to map the fields of self-identified expertise by the panelists and provide a matrix of results. Details of the methodological approach are available elsewhere (Guillemot et al., 2024) [14].

## Results

Twenty-two medical students participated in two Delphi panels between November 2021 to February 2022. This number represents 5% of USFQ medical students (n = 450; margin of error = 8%; confidence level = 90%). Panelists were aged 19–37 years (22 years on average) and were predominantly female (n = 13, one participant preferred not to report their gender). Most researchers self-identified as having a mixed ethnic background (n = 21), and one participant self-identified as ‘Quichua’ (n = 1), an Ecuadorian Indigenous ethnicity. Panel 1 included nine students in their 5th and 6th years, whose main learning methodology was problem-based learning or who attended their rotating internship the year before COVID-19 isolation. Panel 2 included thirteen students from 2nd to 4th year that had just started problem-based learning methodology during the pandemic or were preclinical students. Both panels executed the Delphi methodology simultaneously and independently. No panelist withdrew from the study.

Twenty-two questions as elucidated in ‘Table 1‘ were autonomously formulated by the panelists, as they agreed to have an equal number of questions as there were panelists. We categorized these questions into the following categories: ‘adaptations and innovations’ (twelve questions), ‘assessment and evaluation’ (three questions), ‘virtual clinical practice’ (eight questions), ‘time management’ (six questions), and ‘mental health’ (seven questions). We referred some statements to several categories depending on their approach; hence the number of questions in all categories is greater than the total number of questions. Panel 1 yielded seven higher consensus questions and two mid-consensus questions. In Panel 2, six questions reached a higher consensus and seven a mid-degree of consensus. Neither of the panels yielded lower consensuses.

**Table 1:** Delphi questionnaire and categorization.

Panels	Delphi Question	Category	Degree of consensus	Consensus statement code
**1**	What are the advantages of studying online within a Medicine career?	Adaptations and innovations/Time-management	High (>9/10)	Consensus Statement 1.3.1
	What are the disadvantages of studying online within the Medicine career?	Adaptations and innovations/Virtual clinical practice/Time-management/Mental health	High (>9/10)	Consensus Statement 1.3.2
	Do you think it is necessary to reinforce the subjects taken online during the pandemic, before the students start the rotating internship? Justify your answer	Adaptations and innovations/Virtual clinical practice	High (>9/10)	Consensus Statement 1.3.3
	Do you consider that the professional performance that medical students will provide would be affected by the lack of internships during the pandemic? Justify your answer	Virtual clinical practice	Mid (>7-9/10)	Consensus Statement 1.3.4
	How could the online education system be improved to support the mental health wellness of medical students?	Time-management/Mental health	High (>9/10)	Consensus Statement 1.3.5
	Do you consider that the mental health of medical students was affected during online education in the pandemic? Justify your answer	Time-management/Mental health	High (>9/10)	Consensus Statement 1.3.6
	What is the effectiveness of online practices, through virtual platforms such as iHuman, InSimu, among others?	Adaptations and innovations/Virtual clinical practice	High (>9/10)	Consensus Statement 1.3.7
	How do you consider that virtual classes influenced the management of your time?	Time-management	Mid (>7-9/10)	Consensus Statement 1.3.8
	Do you consider that the amount of study material received in online classes was different compared to face-to-face classes?	Adaptations and innovations	High (>9/10)	Consensus Statement 1.3.9
**2**	For what reasons do you consider that some subjects caused you more stress or disappointment compared to the rest?	Virtual clinical practice/Mental health	High (>9/10)	Consensus Statement 2.3.1
	Do you think that online or virtual simulators were of great use, little or not at all for education during the pandemic? Why?	Adaptations and innovations/Virtual clinical practice	High (>9/10)	Consensus Statement 2.3.2
	Was the use of virtual platforms to take exams together with the various evaluation systems acquired very, little or not at all efficient, considering the problems that some may have presented?	Assessment and evaluation	Mid (>7-9/10)	Consensus Statement 2.3.3
	Were the recordings of the virtual classes very, little or not at all efficient when reviewing material or studying asynchronously?	Adaptations and innovations	High (>9/10)	Consensus Statement 2.3.4
	How long can you retain attention during virtual classes? And what do you consider to be the main distractors?	Adaptations and innovations	High (>9/10)	Consensus Statement 2.3.5
	How has the development of practical skills been in relation to virtual study?	Adaptations and innovations/Virtual clinical practice	Mid (>7-9/10)	Consensus Statement 2.3.6
	What study methods helped you consolidate the concepts seen in class during the pandemic?	Adaptations and innovations	High (>9/10)	Consensus Statement 2.3.7
	Currently, do you consider face-to-face classes to be very, little or not at all necessary? Why?	Assessment and evaluation /Virtual clinical practice	Mid (>7-9/10)	Consensus Statement 2.3.8
	What do you consider to be the biggest challenge of receiving medical classes virtually?	Adaptations and innovations/Mental health	Mid (>7-9/10)	Consensus Statement 2.3.9
	What study tool/method used in the pandemic was able to satisfactorily replace the need for face-to-face meetings?	Adaptations and innovations	High (>9/10)	Consensus Statement 2.3.10
	From your point of view, has the level of academic performance with the virtual modality changed (improved/worsened) compared to the face-to-face modality?	Assessment and evaluation	Mid (>7-9/10)	Consensus Statement 2.3.11
	Do you think that the time saved in transport has been a lot, a little or not at all significant? Have you managed to invest it in something? If the answer is yes, in what?	Time-management/Mental health	Mid (>7-9/10)	Consensus Statement 2.3.12
	What do you think are the most important facts or events to make the decision to leave or stay in the race? (Regardless of your decision)	Mental health	Mid (>7-9/10)	Consensus Statement 2.3.13

### Adaptations and innovations

Panel 1 reported five questions regarding adaptations and innovations and yielded a higher consensus. Students perceive virtual medical education as ‘personalized and didactic’ due to the accessibility and availability of different electronic resources (Consensus Statement 1.3.1). Despite this advantage, panelists agreed that these tools did not substitute face-to-face classes (Consensus Statement 1.3.7) and that the theoretical content learned must be solidified with the practical component (Consensus Statement 1.3.3). Low internet quality and home distractions limited ‘students’ ability to focus’ (Consensus Statement 1.3.2). Additionally, several professors sent more academic workload since they had the ‘erroneous idea that students had more free time during virtual classes’ (Consensus Statement 1.9).

In Panel 2, there were five high-degree Consensus Statements (2.3.2, 2.3.4, 2.3.5, 2.3.7, 2.3.9 and 2.3.10) and one mid-degree Consensus Statement (2.3.6). Panelists considered that the development of practical skills during virtual studies was extremely limited. This occurred particularly in senior years, where ‘patient interaction is required for medical maneuvers, physical examination and clinical history’ (Consensus Statement 2.3.6). Furthermore, class recordings represented the main advantage of this study modality, as they ‘facilitated learning by allowing students to repeat lectures and reinforce concepts not understood’ (Consensus Statement 2.3.4). Additionally, access to recordings ‘enabled them to study independently at their own pace and improve note-taking skills’ (Consensus Statement 2.3.4). However, students did not use class recordings when they perceived their classes based mainly on information published in a book. In this case, they decided to dedicate time towards autonomous learning (Consensus Statement 2.3.9). Lastly, they agreed that the most valuable virtual platforms were those for preparing standardized tests (Consensus Statement 2.3.2).

Students turned to note-taking software, online medical learning platforms, on-demand video libraries, evidence-based clinical resources, flashcard programs, and social media as their main study methods (Consensus Statement 2.3.10). These resources replaced traditional learning strategies such as summaries (Consensus Statement 2.3.7). Additionally, when the use of these resources was insufficient, they joined study groups or attended free student tutorships (Consensus Statement 2.3.7). Nevertheless, students proposed possibly maintaining hybrid or virtual classes in subjects with no practical component (Consensus Statement 2.3.10). Finally, experts agreed that the time they could remain focused during the virtual classes was 25 minutes (Consensus Statement 2.3.5).

### Assessment and evaluation

Three different questions from Panel 2 that reached a mid-degree consensus addressed issues related to assessment and evaluation. The grading system and test-taking strategies changed significantly during the COVID-19 emergency (S4 File).

Regarding test-taking platforms, panelists identified a controversy. While they highlighted that these platforms (mainly Examsoft Ⓡ) were ‘effective in preventing academic dishonesty’, they also stated that effectiveness required relied on external supervision via extra devices, which generated many distractions as microphones and cameras remained on during evaluations. Panelists also emphasized that an extra electronic device was not always available for some students (Consensus Statement 2.3.3).

The main difficulties students faced with using these platforms were ‘damage of software’ and trouble with exam download or upload. Sometimes, the time stipulated for taking the exams was considered insufficient (Consensus Statement 2.3.3). Additionally, panelists stated that ‘tests or different forms of evaluations must be taken in person to preserve academic honesty’ (Consensus Statement 2.3.8). Moreover, they considered that ‘academic performance is no longer reflected in students’ grades, since academic dishonesty and the change in the grading scale (S4 File) shows a decrease in study time’. Others attributed their academic performance results to negative experiences during the pandemic (Consensus Statement 2.3.11).

Although Panel 1 did not discuss this topic and thus no consensus is described in this section, authors agreed that the value of the discussion of panel 2 regarding assessment and evaluation was worthy of an entire category.

### Virtual clinical practice

Panel 1 addressed four questions related to virtual practice. Consensus Statements 1.3.2, 1.3.3, and 1.3.7 received a high-level consensus. Participants considered online practices through virtual platforms as a ‘useful alternative tool to have an initial approach to clinical knowledge, especially in developing clinical history skills’ (Consensus Statement 1.3.7). Nevertheless, the effectiveness of these platforms diminishes by the ‘lack of technical training by professors, teaching assistants, and students’ (Consensus Statement 1.3.2) and ‘the use of sophisticated diagnostics, laboratory or imaging tests that did not exhibit the reality of hospitals in Ecuador’ (Consensus Statement 1.3.7). Additionally, the absence of interaction with real patients is ‘the most challenging event’ for medical students during the pandemic (Consensus Statement 1.3.2). They considered virtual platforms as a complement, but they cannot replace face-to-face practices (Consensus Statement 1.3.7).

The impact of a lack of clinical practices on future professional performance reached a mid-degree consensus (Consensus Statement 1.3.4). Students emphasized that there is high pressure on the medical intern year in terms of performance due to the perception of ‘lacking essential competencies such as the execution of basic medical procedures and social skills’ (Consensus statement 1.3.4). They proposed facultative pre-medical intern year face-to-face workshops to apply the learned theory and develop the practical skills lost during the pandemic. They stated that theoretical content reinforcement is a personal responsibility (Consensus statement 1.3.3).

Panel 2 discussed four questions about virtual practice. Consensus Statements 2.3.1 and 2.3.2 received a high-level consensus, and Consensus Statements 2.3.6 and 2.3.8, a mid-degree consensus. Experts stated that some subjects did not have either virtual or face-to-face practices (Consensus Statement 2.3.1). Additionally, they reported some discrepancies regarding the assessment evaluation system that were unresolved (Consensus Statement 2.3.1). Panelists reported that ‘simulators had a more significant impact and were more efficient in basic sciences such as general biology and molecular biology’ (Consensus Statement 2.3.2). In the first years of medicine, ‘developing practical skills was not such an important problem because most of the classes were theoretical’. However, some subjects with laboratory work were affected somehow (Consensus Statement 2.3.6). Finally, experts concluded that ‘the practical component of all subjects must be strictly face-to-face’ (Consensus Statement 2.3.8).

### Time-management

Panel 1 approached time management in four high-degree consensus questions and one mid-degree consensus question. In some cases, students perceived ‘an increased academic load due to the false belief from professors of excessive free time’ (Consensus Statement 1.3.2). Panelists agreed that unexpected isolation challenged time management, as it was ‘almost impossible to carry out different activities than the academic ones’ (Consensus Statement 1.3.6). However, it was considered that students who had previously acquired organizational skills ‘took advantage of time during virtual classes’ while students who did not, ‘experienced a false sense of excess time, which promoted procrastination’ (Consensus Statement 1.3.8). Additionally, as it was ‘easier to access and participate in a greater number of activities, students experienced an unhealthy over-productivity’ which may lead to burnout syndrome (Consensus Statement 1.3.8).

Panelists concluded that virtual classes later allowed a ‘better time optimization’ as they perceived more time availability between classes. This enabled them to participate in ‘extracurricular activities, study, rest, spend time with family (especially those whose hometown was not the city of Quito)’ and attend ‘free national and international medical conferences and virtual courses’ (Consensus Statement 1.3.1). Students reported increased free time by limited time waste, including eliminating everyday activities such as commuting (Consensus Statement 1.3.8). Experts suggested creating a specific time in class schedules dedicated to ‘homework and class objectives, to avoid burnout’ (Consensus Statement 1.3.5).

Panel 2 discussed time management in one question that acquired a mid-degree consensus. Panelists stated that students who used to spend ‘up to three hours in transportation’ before isolation were the ones that could benefit from saving time and could invest this extra time in ‘eating, exercising, resting, and spending time with family’. On the other hand, students who used to live near the university ‘did not save much time’. This panel also suggested that timesaving was not a determinant of passing or failing a class, as this depends on organizational skills (Consensus Statement 2.3.12).

### Mental health

In panel 1, three questions addressed issues related to mental health, and all achieved a high consensus. Experts recognized medicine as a ‘curriculum with a high prevalence of mental health difficulties’ (Consensus Statement 1.3.6). Factors that contributed to this problem and challenged students’ confidence in their learning process included the non-compliance with class schedules and the lack of hospital practices. Additional stressors were socio-economic concerns, lack of effective communication with lecturers (Consensus Statements 1.3.2), home and online distractions, and using a single space for multiple activities (Consensus Statements 1.3.6). They agreed that adherence to class schedules and restricting academic activities during weekends would improve the balance between personal and professional life (Consensus Statement 1.3.5). Students proposed the creation of free psychological counseling exclusive to the School of Medicine, as well as workshops to cover mental health education techniques available for lecturers and students (Consensus Statement 1.3.5).

Regarding panel 2, four questions addressed mental health. Consensus Statement.2.1 received a high consensus, while Consensus Statements 2.3.9, 2.3.12, and 2.3.13 reached mid-degree consensus. Students considered lecturers’ negative attitudes, limited access to technological tools, and low-quality internet networks as causes of stress and disappointment (Consensus Statement 2.3.1). They reported being impacted by lack of social interaction, difficulty in self-learning, quality of classes, lack of lecturer adaptability, and insufficient support from faculty authorities regarding the significant challenges of receiving virtual medicine classes (Consensus Statement 2.3.9). Experts also stated that ‘avoiding vehicular traffic had a positive impact on mental health’ (Consensus Statement 2.3.12). Furthermore, students discussed the most important elements in deciding to leave or stay in medical school: university and curriculum cost, the feeling of not experiencing a real medical education, and the economic crisis (Consensus Statement 2.3.13). Elements that contributed positively to the student’s overall mental health were personal motivation, time management skills, financial aid, and support by family, friends, and lecturers (Consensus Statement 2.3.13).

## Discussion

This study on participatory Delphi panels conducted at the USFQ School of Medicine in Quito, Ecuador, illustrates the successes and challenges of the sudden transition into a virtual education model caused by the COVID-19 pandemic for approximately two years. We synthesized the successes and challenges of virtual academic training into the categories of adaptations and innovations, assessment and evaluation, virtual clinical practice, time management, and mental health, based on high and medium-grade consensuses obtained from second to sixth-year medical students. Our study lays out solutions to academic deficiencies, fosters student contributions to the design of the medical curricula, and demonstrates how to promote the active involvement of students in topics related to their medical education and research. In addition, this study lays the foundation for post-COVID medical education reforms, where positive lessons associated with distance-learning may be implemented outside a pandemic context.

### Lessons and implications for future medical education strategies

Virtual education strategies offer students a modern approach to learning medicine. Study methods changed drastically as students switched to a variety of medical education platforms, note-taking software, on-demand video libraries for standardized exams, question banks, evidence-based clinical resources, flashcard programs, and social media, which prompted autonomous, personalized and didactic education [15]. In this context, the flipped classroom approach, where knowledge acquisition occurs promptly outside of the classroom and reserves contact with lecturers for group work or questions, left behind lecture-based education [16]. However, the access, use and impact of these online tools differed between academic years [4]. While the work to analyze the lessons associated with the fundamental educational shift imposed by the COVID-19 pandemic have advanced, years will be needed to truly measure its impact and long-term consequences.

This study, for its results, its methodological approach and its socioeconomic context has implication for practice in medical faculties. The outcome highlighted in this study should be considered as universities undergo post-COVID-19 reforms. As many universities prone “going back to normal”, we urge for universities to learn for the lessons learn and retain valuable adaptations, which occurred during the pandemic. As we show, students did not perceived themselves as worse-off in every aspects imposed by the pandemic. The methodological approach, in its participatory fashion, shows that students can actively, productively and academically participate in the development of macro and micro-curricular modifications. With adequate methods and mentoring, students are a key element to the improvement of education and reform. Finally, with regards to the socioeconomic context of this study, while we acknowledge the privilege nature of the academic environment of Universidad San Francisco de Quito, our study suggests that innovation and inspiration for change can proactively and participatively come from the Global South, as opposed to the often encountered Global North to Global South flow of change and reform [17].

### Online education during preclinical years

Multiple studies analyzed even before the pandemic whether medical education during the preclinical years should include in-person classes or focus on virtual methodologies [18]. A study of online learning attitude and computer literacy found that students who had attended online courses reported higher computer literacy and ability to use and search for information than those who had not [19]. Our study shows that virtual or hybrid classes could be retained in subjects where no practical components are required, since these exhibited important benefits associated with time optimization and improvement of study habits [20]. Preclinical students pointed out lecture recordings as the greatest advantage of virtual classes, for these prompted autonomous learning, as students got to decide to attend a particular class or venture into virtual resources that would allow them to acquire theoretical knowledge of their subjects. In addition, this group of students emphasizes that virtual simulators can have a positive impact on basic science subjects, so their permanence as a study tool should be evaluated during the design of medical curricula [21]. Research findings from a questionnaire survey among medical students underscored the benefits of blended learning, such as its flexibility in accommodating diverse learning styles and schedules, facilitation of deeper knowledge acquisition, and cost-effectiveness [22]. However, literature on the implementation of blended learning in resource-limited countries remains scant [23]. While it holds promise in bridging educational disparities for students in remote or underserved areas, challenges including limited access to technology, internet connectivity issues, and cultural readiness may impede its successful implementation [24]. Therefore, comprehensive assessments of socioeconomic contexts and institutional readiness are imperative prior to adoption [25].

### Test taking strategies

Students from earlier years were the only ones who addressed the subject of assessment and evaluation. They proposed that academic evaluations should be kept preferably in-person to avoid cheating, as exam-taking platforms were associated with over supervision, excess noise, and the use of multiple electronic devices. Academic dishonesty and the changes in grading scale may have contributed to the diminishing of studying time. However, strategies to improve virtual evaluations should focus on comparing the use of exam software in combination with standardized questions.

### Online education during clinical years

Panelists from advanced years agreed that virtual tools are an alternative to start approaching the clinical environment; nevertheless, they cannot replace face-to-face classes and practice. On one hand, the availability of a variety of electronic resources and online events, along with time management skills when present, allowed students to participate in world-class projects and classes, enroll in extracurricular activities, implement innovative study habits and obtain individualized learning methodologies. On the other hand, there are strong concerns regarding the lack of essential skills to perform simple procedures on patients among students that missed practice due to the COVID-19 isolation. Similar results have been reported in a multicenter survey of final-year medical students in Belgium, which revealed that more than half of the participants felt the impact of the pandemic on their education and feared consequences for their future academic performance [26]. To compensate for missed clinical practices, students propose face-to-face workshops before the medical intern year. This may motivate universities to increase clinical exposure during the first years of their career by implementing innovative strategies such as student participation in telehealth, group-based interprofessional education (IPE), and electronic health record training [27]. By embracing telemedicine within their training, medical students are better poised to address the evolving needs of patients, regardless of geographic limitations, thereby fostering more inclusive and effective healthcare practices, providing opportunities for interdisciplinary learning and collaboration, improvement of communication skills and fostering a team-based approach to patient care [28, 29].

### Health, leisure and well-being

Although the optimization of time and participation in extracurricular and leisure activities had a positive impact, there is a general negative perspective on mental health in medical students. No significant differences were found between mental health perceptions of pre-clinical and clinical students in panels one and two, respectively. This contrasts with some studies that found greater psychological stress in students in the pre-clinical stage [30, 31]. Panelists perceived an increased academic workload due to the false perception of excessive free time, although increased free time is also reported. Prior organizational skills were fundamental during the isolation as over productivity is described in students who enrolled on a greater number of activities and took advantage of time, and lack of time management skills was associated with procrastination. Similar results were reported in a study that showed that digital learning increases levels of depersonalization, one component of burnout syndrome especially for senior medical students due to the lack of clinical exposure before qualification as junior doctors [32]. Similar to our results, some studies reported that during virtual education, lack of social, physical and mental support from institutions and peers may have prevented students from learning [33–35]. Furthermore, psychological stress was prevalent in students with poor internet connection according to a survey in nine countries as well as in our study. Experts recommend that ensuring a high-speed internet connection could imply a better mental health status of students [31]. Lastly, as the pandemic triggered previous mental health problems and burnout syndrome, universities must offer workshops on the early identification of mental health disorders and easily accessible mental health services [36, 37].

### The role of medical schools

As virtual tools are valuable for their efficacy, improvement of quality and time of study, it should be a priority for medical schools to assess the most demanded platforms used by their students and ensure that these are always available. Based on the consensus reported by the participants of this study, we recommend virtual medical education platforms that integrate standardized exam preparation, question banks and acquisition of medical knowledge. To ensure accessibility, especially in resource-limited countries, schools could venture into mobile learning platforms, social media, open educational resources, offline interactive online modules, or virtual classrooms [38, 39]. The use of these alternatives may encourage healthier lifestyles in medical students since it may balance academic and personal life [8], increase the quality of study and avoid cheating.

Finally, emphasizing that virtual classes prompted advantages in the acquisition of theoretical knowledge, a final implication of the consequences of the COVID-19 pandemic in medical education will rely on universities’ strategies to compensate for the practical integration of such effort [20].

### Strengths

USFQ is the first and only university in Ecuador and one of the few universities in the Global South to consider students’ perception of medical education during the COVID-19 emergency using a fully participatory approach. Considering that COVID-19 pandemic represents a unique event in the world, the expertise of panelists based on their first-hand participation in terms of education may set a precedent for future interventions in the design of contingency protocols in universities in case of emergency situations as well as establish new rules for future curriculum. Additionally, as we included students from different years and diverse ethnic and gender groups, there is a wide representation of the medical student community of this university. The results of participatory Delphi panels point out that students are capable of accounting for various viewpoints involving their learning requirements and, thereby, offer rational and productive policy suggestions. This further confirms our previous work [8].

### Limitations

Although the participatory approach may hamper data quality due to the lack of prior experience of Delphi methodology [40] of authors we believe that the advantages outweigh the risks. In the specific context of this study, it was paramount to involve students of the faculty to enhance the quality of the data collected, which was perceived as a greater advantage. To mitigate this bias, participants had the opportunity to conduct Delphi methodology in their native language, thereby lessening communication bias, although certain elements may have been lost in the translation process.

Little research has been conducted regarding the educational impact on Ecuadorian public and private university students due to the COVID-19 pandemic [41]. There is a potential concern for selection and cultural biases since USFQ socio-economic context differs from most other Ecuadorian institutions and makes this study a representation of a focal reality instead of a comparable response of other Ecuadorian universities to the COVID-19 pandemic. Therefore, the results of this study are only generalizable to certain extents. While participatory methodologies traditionally involve a restricted panelist cohort, future studies could encompass broader and more heterogeneous panels to enhance the inclusivity of diverse student demographics, including other universities [42].

The panelists’ responses could have been influenced by social desirability bias. While we promoted the anonymity of the participants, panel leader knew which student submitted each response. In addition, due to the relative small size of the faculty and social bonds between many participants, it is reasonable to assume that some participants knew who other participants were and who were part of their panel. Cross-university panels could improve the confidentiality of responses [40, 43]. While it is noteworthy that the panelists were involved in design and response of the Delphi questions, there may be limitations about preconceptions or anticipated responses, including confirmation bias. It is crucial to highlight, however, that the study’s findings are grounded in a final consensus, derived through a numeric-graded synthesis. This synthesis resulted from thorough independent discussions among all panelists across three rounds, mitigating the impact of any initial preconceptions. Additionally, it’s important to note that the methodology’s design was undertaken by students who were not part of the development of the Delphi exercises. This separation ensured a degree of impartiality in the overall study design and execution.

## Conclusions and recommendations

We recommend a balanced educational approach that combines the advantages of virtual learning with crucial elements of in-person instruction. This includes the retention of virtual classes for theory-focused subjects, utilizing class recordings for independent study, and adopting hybrid class modalities for practical skills development, especially in senior years. Recognizing the effectiveness of test-taking platforms in preventing academic dishonesty but acknowledging their dependence on external supervision, we recommend in-person tests to uphold academic integrity, reduce distractions, and minimize the need for extra electronic devices during evaluations. Although virtual simulator platforms do not replicate real hospital scenarios completely, they are valuable for the introduction to clinical knowledge in early career stages and should be completed with facultative practical in-person workshops during all training years. Our study emphasizes the positive impact of virtual classes on balancing academic demands and personal well-being, particularly for students with strong organizational skills. To enhance this balance, we suggest promoting time-management practices and encouraging students to engage in extracurricular activities, hobbies, and adequate rest. Comprehensive strategies for mental health suggested in our study include class schedule adherence, limiting weekend academic activities, and introducing exclusive psychological counseling and mental health education workshops for the School of Medicine.

## Supporting information

S1 Video Campaign recruitment. It was delivered via institutional email and social media.
(DOCX)

S2 Video Participatory Delphi methodology training session video. It was available both synchronously and asynchronously.
(DOCX)

S1 File USFQ and school of medicine sociodemographic profile. (DOCX)

S2 File Code of honor and coexistence of Universidad San Francisco de Quito USFQ. (DOCX)

S3 File Delphi enrollment examination. It consisted of a google form with ten methodology questions described during the training session.
(DOCX)

S4 File Grading system changes during COVID-19 pandemic at Universidad San Francisco de Quito (USFQ). (DOCX)
