# Supplementary material for: COVID-19: Medical education from the point of view of medical students using the participatory Delphi method
Source: PLoS One. 2024 Jul 5;19(7):e0297602. doi: 10.1371/journal.pone.0297602 (PMC11226019; doi:10.1371/journal.pone.0297602)
Supplement: S1 Video — It was delivered via institutional email and social media. (DOCX) [file pone.0297602.s001.docx]

**S1 Video. Campaign recruitment.** It was delivered via institutional email and social media.

<https://www.instagram.com/p/CT3MmCnl7Ox/>
