# Supplementary material for: COVID-19: Medical education from the point of view of medical students using the participatory Delphi method
Source: PLoS One. 2024 Jul 5;19(7):e0297602. doi: 10.1371/journal.pone.0297602 (PMC11226019; doi:10.1371/journal.pone.0297602)
Supplement: S2 Video — It was available both synchronously and asynchronously. (DOCX) [file pone.0297602.s002.docx]

**S2 Video. Participatory Delphi Methodology Training session video.** It was available both synchronously and asynchronously.

<https://www.youtube.com/watch?v=YMBr2TaD8lA&ab_channel=ISYN>
