# Supplementary material for: COVID-19: Medical education from the point of view of medical students using the participatory Delphi method
Source: PLoS One. 2024 Jul 5;19(7):e0297602. doi: 10.1371/journal.pone.0297602 (PMC11226019; doi:10.1371/journal.pone.0297602)
Supplement: S1 File — (DOCX) [file pone.0297602.s003.docx]

**S1 File. USFQ and School of Medicine Sociodemographic profile.**

Ecuador proposes a medical education system that includes 24 medical schools divided between public, private/self-financed, and private/co-financed models (Estrella Porter et al. 2020). All universities offer a 6-year program to obtain an undergraduate medical doctor degree. At Universidad San Francisco de Quito (USFQ), a liberal arts private/self-financed university located in Quito, Ecuador, the medical curriculum is founded on basic scientific Socratic discussions during the first and second years. Third year students transition into clinical sciences with a problem-based learning approach and hospital externships. The medical intern year takes place during the sixth year of the curriculum a. At USFQ most students belong to medium-high socio-economic levels.

Ecuador, part of the Andean countries, is located northwest of South America with a population of 17.6 million. In the last decades, the medical education system in Ecuador has grown and changed, with currently 24 medical schools divided into public, private/self-financed, and private/co-financed models [(Estrella Porter et al. 2020)](https://sciwheel.com/work/citation?ids=12549425&pre=&suf=&sa=0). Universidad San Francisco de Quito USFQ is a liberal arts private/self-financed university located in Quito, Ecuador. Founded in 1988 USFQ has become the national leader in higher education, research, creativity, and the unique liberal arts University in the region, reshaping the way research and education have been done in Ecuador.

The composition of the student body at USFQ is diverse in gender, identity, and ethnicity. Nowadays the population of undergraduate and graduate students is around 9,000 students. Intersectionality [(Crenshaw 1989)](https://sciwheel.com/work/citation?ids=12808992&pre=&suf=&sa=0) plays an important role when we analyze the composition of the community, as the different aspects of a person from their different aspects (social, political, economic, ethical, and cultural) combined might influence their experiences and create different scenarios of discrimination and access to privilege. Starting from the concept of intersectionality from a gender layer, the community is divided between 52% of them being women and 48% men, this gender division might influence in both positive and negative ways the experience and challenges faced by the students, affecting their intimacy within their classes and years at USFQ. From an economic layer, 50% of the community is awarded scholarships or academic loans, and to maintain them this student should follow some guidelines that include: academic records (GPA of 3.2 or more), and disciplinary development (not disciplinary faults) as the main requirements. These conditioners determine the experience and development of this student during his/her years at USFQ but also exemplify the way intersectionality works and can determine the conditions and privilege of an individual in a diverse community like USFQ.

On the ethnic layer, USFQ has more than 14 indigenous nationalities and historically discriminated populations within their students, representing 8% of the total population. The historical and factual inequalities in access to education, communication, and health to name three aspects, might affect the performance of these students. Even though USFQ has different programs and actions to equate these harsh conditions as a result of historical discrimination and exclusion, the effects are still visible.

The functional diversity or disability layer is another element to take into consideration when we analyze the composition of the academic community in USFQ, as the only university in Ecuador that actively is working for the inclusion of people with functional diversity or disabilities. 3% of the students have any kind of disability or functional diversity, being another layer that is influencing the experience and performance of students that are part of this community. With this analysis, we show the diversity of the community and the different challenges and variables we took into consideration in the research design, process, and analysis. A glance at the different layers of intersectionality present can portray a more accurate picture of USFQ.

Medical curricula are undergraduate degrees and last six years in the university's medical school:

**
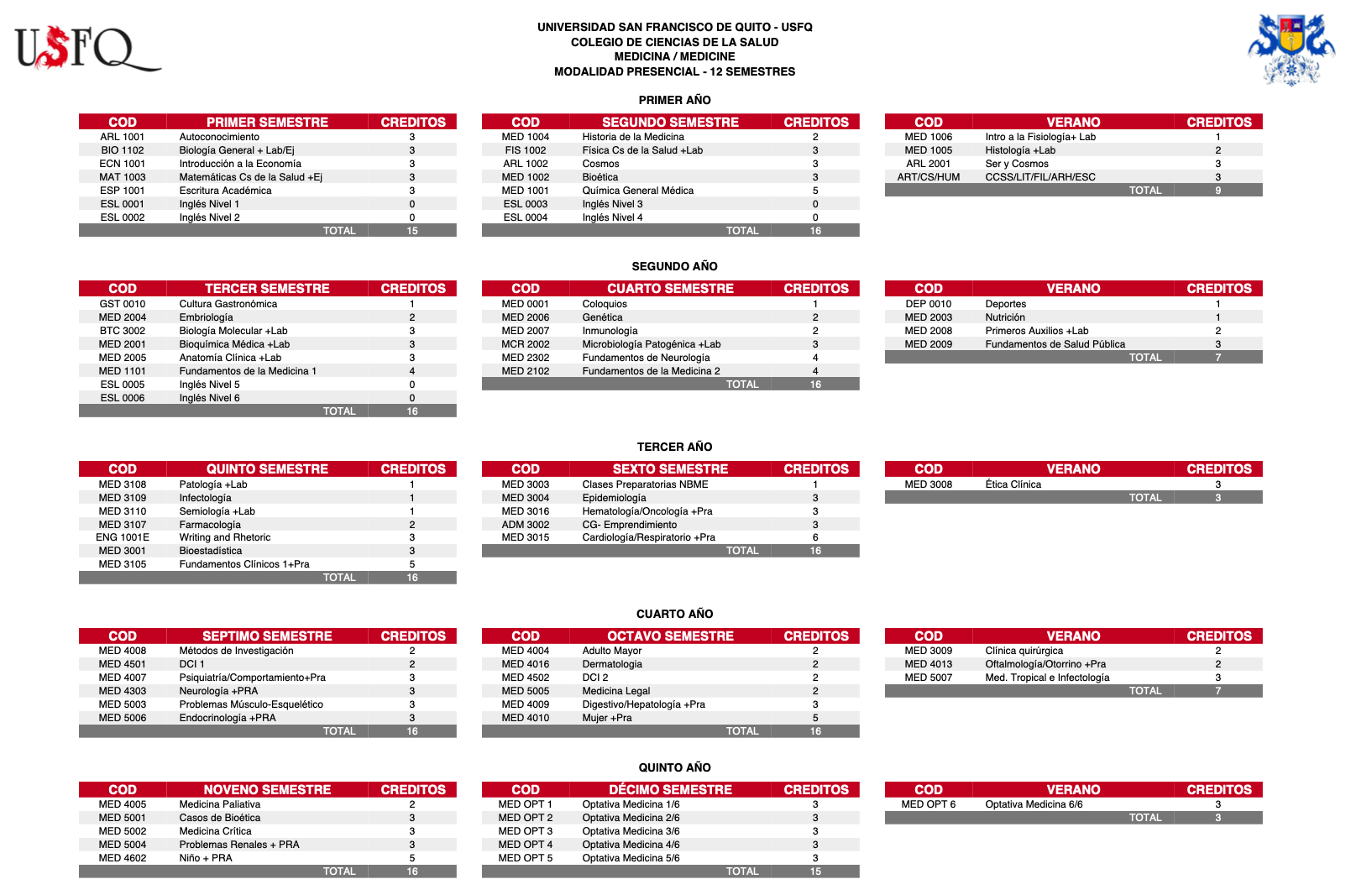
**

**
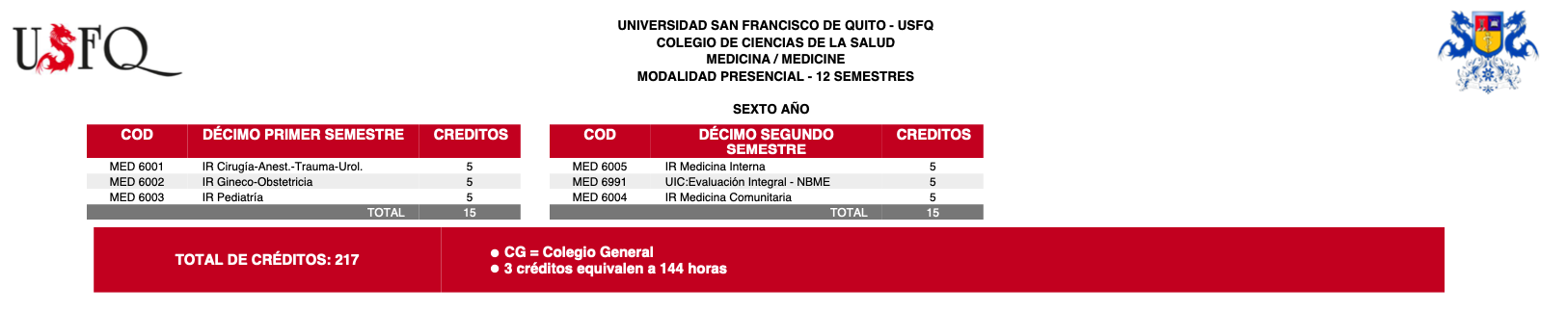
**

At USFQ, students have hospital externships based on actual patient learning since the third year of their career. The rotating internship in Medicine takes place in the sixth year of the career. It aims to consolidate the competencies of the general doctor within a hospital setting and under the supervision of a resident or attending physician.

Over 21 months, the USFQ grading scale changed due to the health emergency. The equivalence of the range of grades scores to letter grades decreased by 3 points for each parameter. For instance, if the letter A was previously equivalent to a range of 91 to 100 points, now the range will broaden from 89 to 100 points.

The composition of the medical student body at USFQ is diverse in gender, identity, and ethnicity, resembling the composition of the whole university. Nowadays the population of undergraduate students is 677 people approx., representing 7% of the total population of the university. From a gender layer, the community is divided between 52% of them being women and 48% men, this gender division is similar to the general population at USFQ. As we established before this might influence the experience and challenges faced by the students, affecting their intimacy within their classes and years at USFQ.

On the ethnic layer, the USFQ School of Medicine has a 6% of its students self-identify as part of one of the 14 indigenous nationalities including African Ecuadoreans and Indigenous from the Sierra and Amazonian Region. At the functional diversity or disability layer the composition of the academic community in the medical student body, represents 1,2% of the students who have any kind of disability or functional diversity. All these layers are influencing the experience and performance of students that are part of the USFQ community.
