# Supplementary material for: COVID-19: Medical education from the point of view of medical students using the participatory Delphi method
Source: PLoS One. 2024 Jul 5;19(7):e0297602. doi: 10.1371/journal.pone.0297602 (PMC11226019; doi:10.1371/journal.pone.0297602)
Supplement: S2 File — (DOCX) [file pone.0297602.s004.docx]

**S2 File. Code of Honor and Coexistence of Universidad San Francisco de Quito USFQ**

The Code of Honor and Coexistence of Universidad San Francisco de Quito USFQ presents the guiding principle of their university promoting the harmonious and friendly coexistence among its members. All members of the university commit themselves to be honest and to respect the norms of this Code for the common good. Read here for further details: <https://web.archive.org/web/20230116041057/https://www.usfq.edu.ec/en/code-of-honour-and-coexistence>
