# Supplementary material for: COVID-19: Medical education from the point of view of medical students using the participatory Delphi method
Source: PLoS One. 2024 Jul 5;19(7):e0297602. doi: 10.1371/journal.pone.0297602 (PMC11226019; doi:10.1371/journal.pone.0297602)
Supplement: S3 File — It consisted of a google form with ten methodology questions described during the training session. (DOCX) [file pone.0297602.s005.docx]

**S3 File. Delphi enrollment examination.** It consisted of a google form with ten methodology questions described during the training session.

1.- How many rounds, or iterative processes, does the Delphi methodology proposed in the training have?

1. 2
2. 3
3. 4
4. 5

2.- What is a round in the context of a Delphi panel? (more than one correct answer)

1. An element of the iterative process
2. The moment when the final manuscript of a Delphi panel is prepared
3. A part of the Delphi process during which the Delphi questionnaire is sent, answered and synthesized
4. A part of the Delphi process where panelists talk together to choose how to answer the question

3.- Why is the anonymity of the panelists' response in each round in a Delphi panel important? (more than one correct answer)

1. To protect the personal data of panelists
2. To ensure that participants feel free to weigh in without risk of judgment from other panelists
3. To ensure that the intellectual property of all panelists is respected in the event of the creation of a patent
4. To avoid influence between panelists

4.- Choose the correct answer item(s). A Delphi panel is…

1. A divination methodology born in Ancient Greece with bird watching
2. A class to teach scientific inquiry
3. An iterative process with the objective of convergence of opinion
4. A methodology that seeks to develop consensus among experts

5.- The Delphi methodology is a quantitative research methodology

1. True
2. False

6.- The type of question that can be used in the Delphi methodology is very specific. Choose the appropriate question for a Delphi panel from the following options:

1. In what year was radioactivity discovered and by whom?
2. Does God exist?
3. How old are you and what year did you start studying at university?
4. Since when do you think most of the cars in the world will be electric and why?

7.- How many experts do we plan to include in each Delphi panel?

1. 3-5
2. 8-15
3. 25-40
4. 25-150

8.- Choose the example of a common use of Delphi panels in health research

1. Clinical trial
2. Literature study
3. Statistical study of epidemiology
4. Development of clinical recommendation

9.- What is a participatory study?

1. A study that requires a voting process
2. A study on political behavior of the population and its level of political involvement
3. A study that involves the study population as a researcher
4. A study which measures any type of participation

10.- Why is a synthesis notation process included during the Delphi process?

1. To evaluate the quality of the work of the panelists
2. To be able to choose the panelists
3. To measure the level of approval on the synthesis of the panelists
4. To know if it is necessary to include more panelists in the Delphi panel and thus find a consensus

Not graded question: If you are invited to participate in this research study, would you like to be the leader of your participatory panel?

1. Yes
2. No
