# Supplementary material for: COVID-19: Medical education from the point of view of medical students using the participatory Delphi method
Source: PLoS One. 2024 Jul 5;19(7):e0297602. doi: 10.1371/journal.pone.0297602 (PMC11226019; doi:10.1371/journal.pone.0297602)
Supplement: S4 File — (DOCX) [file pone.0297602.s006.docx]

**S5 File. Grading system changes during COVID-19 pandemic at Universidad San Francisco de Quito (USFQ).**

**
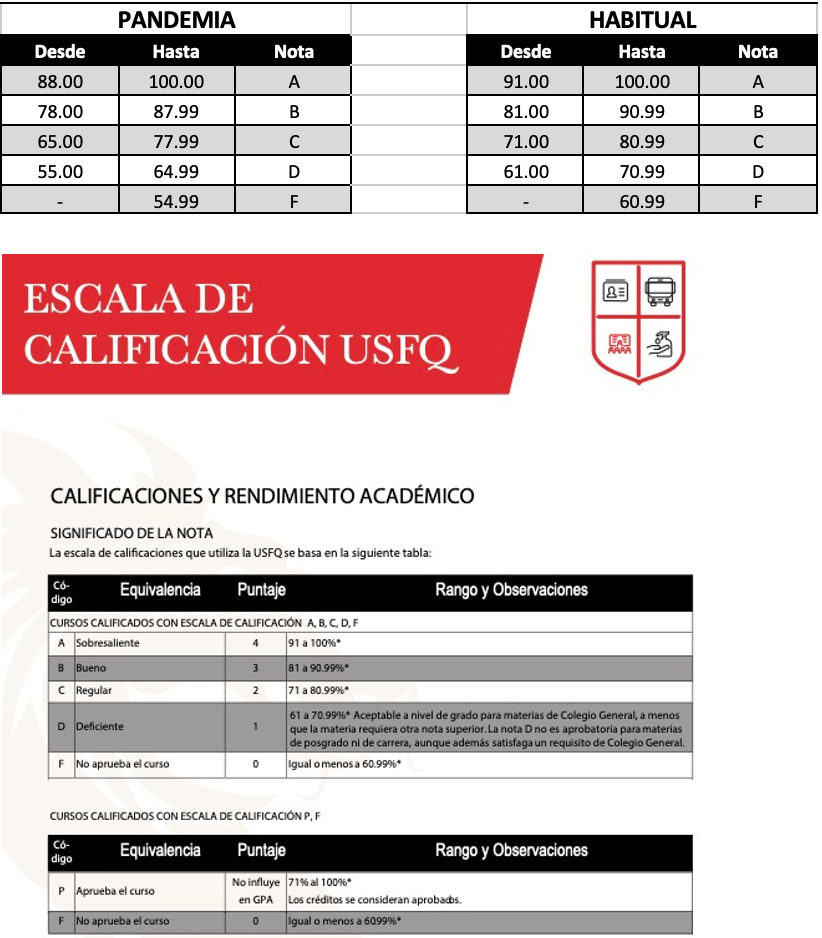
**
