# Supplementary material for: COVID-19: Medical education from the point of view of medical students using the participatory Delphi method
Source: PLoS One. 2024 Jul 5;19(7):e0297602. doi: 10.1371/journal.pone.0297602 (PMC11226019; doi:10.1371/journal.pone.0297602)
Supplement: Attachment — Submitted filename: Rebuttal letter.docx [file pone.0297602.s007.docx]

Andrea Gabriela Ortiz Riofrio

Universidad San Francisco de Quito

Diego de Robles s/n, Quito 170901

Cumbaya, Ecuador, 170901

[agortizr00@outlook.com](mailto:agortizr00@outlook.com)

Friday, March 15th, 2024

Dear Dr Naeem Mubarak, PhD

Academic Editor PLOS ONE,

Subject: Rebuttal to Reviewer Comments on Manuscript [PONE-D-23-42944]

I hope this letter finds you well. I am writing in response to the comments provided by the reviewers regarding our manuscript titled "COVID-19: medical education from the point of view of medical students using the participatory Delphi method," which was submitted for consideration in PLOS ONE.

First and foremost, we would like to express our gratitude to you as well as to the reviewers for your thorough evaluation and constructive feedback on our work. We appreciate the time and effort you have dedicated to assessing our manuscript.

We have carefully reviewed the comments provided by the reviewers and have made the necessary revisions to address your concerns. Below, we provide detailed responses to each of the reviewers' comments:

**Sample Size: The number of participants (22 medical students) might be considered relatively small. A larger and more diverse sample would strengthen the generalizability of the findings. Consider addressing this limitation or discussing its impact on the study's external validity.**

[Response to Reviewer 1's Comments]: Although we described that a Delphi exercise typically unites 8–20 experts and that our sample size included only 5% of the USFQ medical school population (an expected number of participants), we acknowledge that further justification was required, thus we added an additional citation in the second paragraph of the methods section [11].

To further address this comment we included in the second paragraph of the limitations section the following: Therefore, the results of this study are only generalizable to certain extents. While participatory methodologies traditionally involve a restricted panelist cohort, future studies could encompass broader and more heterogeneous panels to enhance the inclusivity of diverse student demographics, including other universities [41].

**Methodological Rigor: While the participatory Delphi method is innovative, a more detailed explanation of the steps taken during the recruitment campaign, training, and constitution of Delphi panels would enhance the methodological rigor. This would assist readers and future researchers in replicating the study.**

[Response to Reviewer 1's Comments]: Given the significance and applicability of the Delphi methodology within the context of medical student research, we have dedicated a separate manuscript delineating the methodological intricacies entailed in the design and execution phases of our study, which remains unpublished at present, as its submission first awaits the acceptance of the results paper. We believe that the outline provided within the current manuscript offers a comprehensive overview of the procedural framework. Coupled with the supplementary materials, we are confident that these resources will serve as invaluable references, inspiring prospective researchers to replicate and build upon our study in future endeavors. A thorough description of our methodological approach would have required a methodological section we judged too long.

**Limitations Acknowledgment: The article lacks explicit discussion of the limitations inherent in the study. Addressing potential biases, constraints, or challenges faced during the research process would contribute to the transparency and credibility of the study.**

[Response to Reviewer 1's Comments]:

We broaden the limitations section by discussing methods implemented to mitigate communication, selection, cultural, social desirability, and confirmations bias.

In the first paragraph we discussed communication bias: In the specific context of this study, it was paramount to involve students of the faculty to enhance the quality of the data collected, which was perceived as a greater advantage. To mitigate this bias, participants had the opportunity to conduct Delphi methodology in their native language, thereby lessening communication bias, although certain elements may have been lost in the translation process.

In the second line of the second paragraph we included: There is a potential concern for selection and cultural biases since USFQ socio-economic context differs from most other Ecuadorian institutions and makes this study a representation of a focal reality instead of a comparable response of other Ecuadorian universities to the COVID-19 pandemic.

We added a third paragraph to discuss social desirability bias: The panelists' responses could have been influenced by social desirability bias. While we promoted the anonymity of the participants, the panel leader knew which student submitted each response. In addition, due to the relatively small size of the faculty and social bonds between 25 many participants, it is reasonable to assume that some participants knew who other participants were and who were part of their panel. Cross-univeristy panels could improve the confidentiality of responses [42] [39].

In the sixth line of the third paragraph we discussed confirmation bias: While it is noteworthy that the panelists were involved in design and response of the Delphi questions, there may be limitations about preconceptions or anticipated responses, including confirmation bias. It is crucial to highlight, however, that the study's findings are grounded in a final consensus, derived through a numeric-graded synthesis. This synthesis resulted from thorough independent discussions among all panelists across three rounds, mitigating the impact of any initial preconceptions. Additionally, it's important to note that the methodology's design was undertaken by students who were not part of the development of the Delphi exercises. This separation ensured a degree of impartiality in the overall study design and execution.

Challenges and constraints are further explained in the methodology manuscript previously mentioned.

**Further Discussion on Implications: The article briefly touches upon the consensuses reached but could benefit from a more in-depth discussion of the implications of these consensuses. How can these findings inform future medical education strategies, especially in resource-limited contexts?**

[Response to Reviewer 1's Comments]: We broaden the implications of future medical education in resource-limited contexts in different subheading of the discussion section:

We rename the Lessons subheading to Lessons and implications for future medical education strategies

We included a second paragraph in the subheading Lessons and implications for future medical education strategies: This study, for its results, its methodological approach and its socioeconomic context has implications for practice in medical faculties. The outcome highlighted in this study should be considered as universities undergo post-COVID-19 reforms. As many universities are prone to "going back to normal", we urge universities to learn from the lessons learned and retain valuable adaptations, which occurred during the pandemic. As we show, students did not perceive themselves as worse-off in every aspect imposed by the pandemic. The methodological approach, in its participatory fashion, shows that students can actively, productively and academically participate in the development of macro and micro-curricular modifications. With adequate methods and mentoring, students are a key element to the improvement of education and reform. Finally, with regards to the socioeconomic context of this study, while we acknowledge the privilege nature of the academic environment of Universidad San Francisco de Quito, our study suggests that innovation and inspiration for change can proactively and participatively come from the Global South, as opposed to the often encountered Global North to Global South flow of change and reform [16].

Subheading: Online education during preclinical years, line 14-23: Research findings from a questionnaire survey among medical students underscored the benefits of blended learning, such as its flexibility in accommodating diverse learning styles and schedules, facilitation of deeper knowledge acquisition, and cost-effectiveness[21]. However, literature on the implementation of blended learning in resource-limited countries remains scant [22]. While it holds promise in bridging educational disparities for students in remote or underserved areas, challenges including limited access to technology, internet connectivity issues, and cultural readiness may impede its successful implementation [23]. Therefore, comprehensive assessments of socioeconomic contexts and institutional readiness are imperative prior to adoption [24].

Subheading: Medical education during clinical years, line 16-20: By embracing telemedicine within their training, medical students are better poised to address the evolving needs of patients, regardless of geographic limitations, thereby fostering more inclusive and effective healthcare practices, providing opportunities for interdisciplinary learning and collaboration, improvement of communication skills and fostering a team-based approach to patient care [27,28].

Subheading: the role of medical schools, line 6-9: To ensure accessibility, especially in resource-limited countries, schools could venture into mobile learning platforms, social media, open educational resources, offline interactive online modules, or virtual classrooms [37,38].

**References: The reference list ist very short. The authors should read through relevant academic literature and discuss their results in more depth with prior studies. I would suggest starting with certain landmark articles.**

Thank you for this comment. We included the three articles suggested by the reviewer that correspond to the [30], [34], [36] reference.

We broaden our reference list across the manuscript including a total list of 42.

We have reviewed each reference individually and we confirmed that each of them are valid and have not been rejected.

**The conclusion section of the manuscript should be presented in a single paragraph.**

[Response to Reviewer 2's Comments]

The conclusion section is now presented in a single paragraph: We recommend a balanced educational approach that combines the advantages of virtual learning with crucial elements of in-person instruction. This includes the retention of virtual classes for theory-focused subjects, utilizing class recordings for independent study, and adopting hybrid class modalities for practical skills development, especially in senior years. Recognizing the effectiveness of test-taking platforms in preventing academic dishonesty but acknowledging their dependence on external supervision, we recommend in-person tests to uphold academic integrity, reduce distractions, and minimize the need for extra electronic devices during evaluations. Although virtual simulator platforms do not replicate real hospital scenarios completely, they are valuable for the introduction to clinical knowledge in early career stages and should be completed with facultative practical in-person workshops during all training years. Our study emphasizes the positive impact of virtual classes on balancing academic demands and personal well-being, particularly for students with strong organizational skills. To enhance this balance, we suggest promoting time-management practices and encouraging students to engage in extracurricular activities, hobbies, and adequate rest. Comprehensive strategies for mental health suggested in our study include class schedule adherence, limiting weekend academic activities, and introducing exclusive psychological counseling and mental health education workshops for the School of Medicine.

**Additional editors comments**

We have ensured that our revised version meets PLOS ONE's style requirements, including those for file naming and style templates. We also have included a legend listed in the manuscript after the references list for each of the Supporting Information files. Supporting information files are included as separate files from the original manuscript. We updated our reference list and included reference 11,16,21,22,23,24,27,28,32,33,34,36,37,38,39,41,42.

In addressing the comments, we have made several modifications to the manuscript, including clarifications in the methodology section, revisions to the discussion to better contextualize our findings within the existing literature, expand the reference list and explain the limitations we faced along the process. We believe that these changes have significantly improved the quality and clarity of the manuscript.

We would like to highlight that while we have carefully considered the reviewers' suggestions, we have also exercised our judgment in retaining certain elements of the manuscript that we believe are essential to the integrity and coherence of our research.

We are confident that the revised version of the manuscript meets the standards of PLOS ONE and makes a valuable contribution to the field. We hope that the reviewers will find our responses satisfactory and that they will recommend the acceptance of the manuscript for publication. We welcome any additional comments that reviewers may encounter.

Thank you once again for the opportunity to submit our work to PLOS ONE. We look forward to hearing from you soon regarding the final decision on our manuscript.

Sincerely,

Andrea Gabriela Ortiz

Universidad San Francisco de Quito
