# Supplementary material for: COVID-19: Medical education from the point of view of medical students using the participatory Delphi method
Source: PLoS One. 2024 Jul 5;19(7):e0297602. doi: 10.1371/journal.pone.0297602 (PMC11226019; doi:10.1371/journal.pone.0297602)
Supplement: Peer review history

# Peer review: Decision Letter 0

**DOI:** 10.1371/journal.pone.0297602.r001

**Authors:** Naeem Mubarak

30 Jan 2024

PONE-D-23-42944COVID-19: medical education from the point of view of medical students using the participatory Delphi methodPLOS ONE

Dear Dr. Ortiz Riofrio,

Thank you for submitting your manuscript to PLOS ONE. After careful consideration, we feel that it has merit but does not fully meet PLOS ONE’s publication criteria as it currently stands. Therefore, we invite you to submit a revised version of the manuscript that addresses the points raised during the review process.

Please submit your revised manuscript by Mar 15 2024 11:59PM. If you will need more time than this to complete your revisions, please reply to this message or contact the journal office at plosone@plos.org. When you're ready to submit your revision, log on to https://www.editorialmanager.com/pone/ and select the 'Submissions Needing Revision' folder to locate your manuscript file.

Please include the following items when submitting your revised manuscript:

- A rebuttal letter that responds to each point raised by the academic editor and reviewer(s). You should upload this letter as a separate file labeled 'Response to Reviewers'.
- A marked-up copy of your manuscript that highlights changes made to the original version. You should upload this as a separate file labeled 'Revised Manuscript with Track Changes'.
- An unmarked version of your revised paper without tracked changes. You should upload this as a separate file labeled 'Manuscript'.

If you would like to make changes to your financial disclosure, please include your updated statement in your cover letter. Guidelines for resubmitting your figure files are available below the reviewer comments at the end of this letter.

We look forward to receiving your revised manuscript.

Kind regards,

Naeem Mubarak, PhD

Academic Editor

PLOS ONE

Journal requirements:

When submitting your revision, we need you to address these additional requirements.

1. Please ensure that your manuscript meets PLOS ONE's style requirements, including those for file naming. The PLOS ONE style templates can be found at

https://journals.plos.org/plosone/s/file?id=wjVg/PLOSOne_formatting_sample_main_body.pdf and

https://journals.plos.org/plosone/s/file?id=ba62/PLOSOne_formatting_sample_title_authors_affiliations.pdf

2. We notice that your supplementary figures are uploaded with the file type 'Figure'. Please amend the file type to 'Supporting Information'. Please ensure that each Supporting Information file has a legend listed in the manuscript after the references list.

3. We notice that your supplementary files are included in the manuscript file. Please remove them and upload them with the file type 'Supporting Information'. Please ensure that each Supporting Information file has a legend listed in the manuscript after the references list.

4. Please review your reference list to ensure that it is complete and correct. If you have cited papers that have been retracted, please include the rationale for doing so in the manuscript text, or remove these references and replace them with relevant current references. Any changes to the reference list should be mentioned in the rebuttal letter that accompanies your revised manuscript. If you need to cite a retracted article, indicate the article’s retracted status in the References list and also include a citation and full reference for the retraction notice.

Additional Editor Comments:

The manuscript has a good deal of merit for publication and may be accepted after minor revisions

[Note: HTML markup is below. Please do not edit.]

Reviewers' comments:

Reviewer's Responses to Questions

**Comments to the Author**

1. Is the manuscript technically sound, and do the data support the conclusions?

The manuscript must describe a technically sound piece of scientific research with data that supports the conclusions. Experiments must have been conducted rigorously, with appropriate controls, replication, and sample sizes. The conclusions must be drawn appropriately based on the data presented. 

Reviewer #1: Yes

Reviewer #2: Yes

**********

2. Has the statistical analysis been performed appropriately and rigorously? 

Reviewer #1: Yes

Reviewer #2: N/A

**********

3. Have the authors made all data underlying the findings in their manuscript fully available?

The PLOS Data policy requires authors to make all data underlying the findings described in their manuscript fully available without restriction, with rare exception (please refer to the Data Availability Statement in the manuscript PDF file). The data should be provided as part of the manuscript or its supporting information, or deposited to a public repository. For example, in addition to summary statistics, the data points behind means, medians and variance measures should be available. If there are restrictions on publicly sharing data—e.g. participant privacy or use of data from a third party—those must be specified.

Reviewer #1: Yes

Reviewer #2: Yes

**********

4. Is the manuscript presented in an intelligible fashion and written in standard English?

PLOS ONE does not copyedit accepted manuscripts, so the language in submitted articles must be clear, correct, and unambiguous. Any typographical or grammatical errors should be corrected at revision, so please note any specific errors here.

Reviewer #1: Yes

Reviewer #2: Yes

**********

5. Review Comments to the Author

Please use the space provided to explain your answers to the questions above. You may also include additional comments for the author, including concerns about dual publication, research ethics, or publication ethics. (Please upload your review as an attachment if it exceeds 20,000 characters)

Reviewer #1: Abstract Evaluation:

Strengths:

Relevance and Timeliness: The article addresses a critical aspect of medical education during the COVID-19 pandemic, focusing on the experiences of medical students in a Global South university. Given the global impact of the pandemic on education, this study adds timely and relevant insights.

Innovative Methodology: The use of the participatory Delphi method is commendable, providing a structured approach to gather and analyze opinions from medical students. This methodology appears to be well-implemented and allows for the exploration of diverse perspectives.

Comprehensive Analysis: The article successfully captures a range of aspects related to medical education during the pandemic, including adaptations and innovations, curriculum changes, virtual clinical practice, time management, and mental health. This comprehensive approach enriches the study and provides a holistic understanding of the challenges faced.

Clear Presentation of Findings: The findings are well-organized, with distinct categories for analysis and clear identification of consensuses based on students' academic year. This clarity enhances the reader's understanding of the diverse impacts on different cohorts of students.

Areas for Improvement:

Sample Size: The number of participants (22 medical students) might be considered relatively small. A larger and more diverse sample would strengthen the generalizability of the findings. Consider addressing this limitation or discussing its impact on the study's external validity.

Methodological Rigor: While the participatory Delphi method is innovative, a more detailed explanation of the steps taken during the recruitment campaign, training, and constitution of Delphi panels would enhance the methodological rigor. This would assist readers and future researchers in replicating the study.

Limitations Acknowledgment: The article lacks explicit discussion of the limitations inherent in the study. Addressing potential biases, constraints, or challenges faced during the research process would contribute to the transparency and credibility of the study.

Further Discussion on Implications: The article briefly touches upon the consensuses reached but could benefit from a more in-depth discussion of the implications of these consensuses. How can these findings inform future medical education strategies, especially in resource-limited contexts?

References: The reference list ist very short. The authors should read through relevant academic literature and discuss their results in more depth with prior studies. I would suggest starting with certain landmark articles, such as the following:

- https://doi.org/10.5116%2Fijme.6209.10d6

- https://doi.org/10.1177/10901981211019292

- https://doi.org/10.1108/JMHTEP-03-2021-0035

Conclusion:

In conclusion, the article provides valuable insights into the impact of the COVID-19 pandemic on medical education, employing an innovative participatory Delphi method. While the strengths of the study are evident, addressing the highlighted areas for improvement would enhance the overall quality and applicability of the research. With these considerations, I recommend the publication of this article after revisions to address the mentioned points.

Reviewer #2: Review comments

General comments

I commend the Authors for the well-presented manuscript which also dealt on a topic that is of much relevance to the future of medical education.

I applaud the Authors for their good efforts in presenting the manuscript for publication and request that they conform to Journal guidelines in the presentation of the manuscript. For example, the conclusion section of the manuscript should be presented in a single paragraph.

I recommend that the manuscript should be accepted for publication.

**********

6. PLOS authors have the option to publish the peer review history of their article (what does this mean?). If published, this will include your full peer review and any attached files.

If you choose “no”, your identity will remain anonymous but your review may still be made public.

**Do you want your identity to be public for this peer review?** For information about this choice, including consent withdrawal, please see our Privacy Policy.

Reviewer #1: No

Reviewer #2: **Yes: **EDMUND NDUDI OSSAI

**********

[NOTE: If reviewer comments were submitted as an attachment file, they will be attached to this email and accessible via the submission site. Please log into your account, locate the manuscript record, and check for the action link "View Attachments". If this link does not appear, there are no attachment files.]

While revising your submission, please upload your figure files to the Preflight Analysis and Conversion Engine (PACE) digital diagnostic tool, https://pacev2.apexcovantage.com/. PACE helps ensure that figures meet PLOS requirements. To use PACE, you must first register as a user. Registration is free. Then, login and navigate to the UPLOAD tab, where you will find detailed instructions on how to use the tool. If you encounter any issues or have any questions when using PACE, please email PLOS at figures@plos.org. Please note that Supporting Information files do not need this step.

# Author comment: Author response to Decision Letter 0

**DOI:** 10.1371/journal.pone.0297602.r002

15 Mar 2024

Please refer to rebuttal letter submitted in the Attach Files section

Attachment Submitted filename: Rebuttal letter.docx

# Peer review: Decision Letter 1

**DOI:** 10.1371/journal.pone.0297602.r003

**Authors:** Naeem Mubarak

5 Apr 2024

COVID-19: medical education from the point of view of medical students using the participatory Delphi method

PONE-D-23-42944R1

Dear Dr. Andrea Gabriela Ortiz Riofrio,

We’re pleased to inform you that your manuscript has been judged scientifically suitable for publication and will be formally accepted for publication once it meets all outstanding technical requirements.

Within one week, you’ll receive an e-mail detailing the required amendments. When these have been addressed, you’ll receive a formal acceptance letter and your manuscript will be scheduled for publication.

An invoice will be generated when your article is formally accepted. Please note, if your institution has a publishing partnership with PLOS and your article meets the relevant criteria, all or part of your publication costs will be covered. Please make sure your user information is up-to-date by logging into Editorial Manager at Editorial Manager® and clicking the ‘Update My Information' link at the top of the page. If you have any questions relating to publication charges, please contact our Author Billing department directly at authorbilling@plos.org.

If your institution or institutions have a press office, please notify them about your upcoming paper to help maximize its impact. If they’ll be preparing press materials, please inform our press team as soon as possible -- no later than 48 hours after receiving the formal acceptance. Your manuscript will remain under strict press embargo until 2 pm Eastern Time on the date of publication. For more information, please contact onepress@plos.org.

Kind regards,

Naeem Mubarak, PhD

Academic Editor

PLOS ONE

Additional Editor Comments (optional):

The manuscript holds a good deal of merit and may be accepted for publication

Reviewers' comments:

Reviewer's Responses to Questions

**Comments to the Author**

1. If the authors have adequately addressed your comments raised in a previous round of review and you feel that this manuscript is now acceptable for publication, you may indicate that here to bypass the “Comments to the Author” section, enter your conflict of interest statement in the “Confidential to Editor” section, and submit your "Accept" recommendation.

Reviewer #1: All comments have been addressed

Reviewer #2: All comments have been addressed

**********

2. Is the manuscript technically sound, and do the data support the conclusions?

The manuscript must describe a technically sound piece of scientific research with data that supports the conclusions. Experiments must have been conducted rigorously, with appropriate controls, replication, and sample sizes. The conclusions must be drawn appropriately based on the data presented. 

Reviewer #1: Yes

Reviewer #2: Yes

**********

3. Has the statistical analysis been performed appropriately and rigorously? 

Reviewer #1: Yes

Reviewer #2: N/A

**********

4. Have the authors made all data underlying the findings in their manuscript fully available?

The PLOS Data policy requires authors to make all data underlying the findings described in their manuscript fully available without restriction, with rare exception (please refer to the Data Availability Statement in the manuscript PDF file). The data should be provided as part of the manuscript or its supporting information, or deposited to a public repository. For example, in addition to summary statistics, the data points behind means, medians and variance measures should be available. If there are restrictions on publicly sharing data—e.g. participant privacy or use of data from a third party—those must be specified.

Reviewer #1: Yes

Reviewer #2: Yes

**********

5. Is the manuscript presented in an intelligible fashion and written in standard English?

PLOS ONE does not copyedit accepted manuscripts, so the language in submitted articles must be clear, correct, and unambiguous. Any typographical or grammatical errors should be corrected at revision, so please note any specific errors here.

Reviewer #1: Yes

Reviewer #2: Yes

**********

6. Review Comments to the Author

Please use the space provided to explain your answers to the questions above. You may also include additional comments for the author, including concerns about dual publication, research ethics, or publication ethics. (Please upload your review as an attachment if it exceeds 20,000 characters)

Reviewer #1: The authors have adressed all outstanding issued. This is a great article, well done! I look forward to regarding more of the authors work in the future.

Reviewer #2: (No Response)

**********

7. PLOS authors have the option to publish the peer review history of their article (what does this mean?). If published, this will include your full peer review and any attached files.

If you choose “no”, your identity will remain anonymous but your review may still be made public.

**Do you want your identity to be public for this peer review?** For information about this choice, including consent withdrawal, please see our Privacy Policy.

Reviewer #1: No

Reviewer #2: **Yes: **EDMUND NDUDI OSSAI

**********
